# Presence and Implications of Anti‐Angiotensin Converting Enzyme‐2 Immunoglobulin M Antibodies in Anti‐Melanoma‐Differentiation‐Associated 5 Dermatomyositis

**DOI:** 10.1002/acr2.11423

**Published:** 2022-03-01

**Authors:** Christopher A. Mecoli, Akira Yoshida, Julie J. Paik, Cheng Ting Lin, Sonye Danoff, Hironari Hanaoka, Antony Rosen, Lisa Christopher‐Stine, Masataka Kuwana, Livia Casciola‐Rosen

**Affiliations:** ^1^ Johns Hopkins University School of Medicine Baltimore Maryland; ^2^ Nippon Medical School Graduate School of Medicine Tokyo Japan; ^3^ Keio University School of Medicine Tokyo Japan

## Abstract

**Objective:**

Patients with anti‐melanoma‐differentiation‐associated 5 (anti‐MDA5)‐positive dermatomyositis (DM) share several striking similarities to patients with SARS‐CoV‐2. Our objective was to assess the prevalence of anti‐angiotensin converting enzyme‐2 (ACE2) immunoglobulin M (IgM) antibodies, found in patients with severe SARS‐CoV‐2, in two independent anti‐MDA5‐positive DM cohorts.

**Methods:**

Anti‐ACE2 IgM antibodies were assayed by enzyme‐linked immunosorbent assay (ELISA) in two anti‐MDA5‐positive DM cohorts: a predominantly outpatient North American cohort (n = 52) and a Japanese cohort enriched for new‐onset disease (n = 32). Additionally, 118 patients with SARS‐CoV‐2 with a spectrum of clinical severity were tested for anti‐MDA5 antibodies by ELISA.

**Results:**

Five of fifty‐two (9.6%) North American patients and five of thirty‐two (15%) Japanese patients were positive for anti‐ACE2 IgM. In the North American cohort, all five patients with anti‐ACE2 IgM antibodies had proximal muscle weakness, had interstitial lung disease, were significantly more likely to receive pulse dose methylprednisolone (80% vs 30%, *P* = 0.043), and had worse forced vital capacity (median 59% predicted vs 78%, *P* = 0.056) compared with the anti‐ACE2‐IgM‐negative group. In the Japanese cohort, all five anti‐ACE2‐IgM‐positive patients also required pulse dose methylprednisolone, and three of five (60%) patients died. Japanese patients with anti‐ACE2 IgM had significantly worse oxygenation, as defined by a lower partial pressure of oxygen (PaO2)/fraction of inspired oxygen (FiO2) ratio (233 vs 390, *P* = 0.021), and a higher alveolar‐arterial oxygenation gradient (91 vs 23 mm Hg, *P* = 0.024) than the IgM‐negative group.

**Conclusion:**

We describe anti‐ACE2 IgM autoantibodies in two independent cohorts with anti‐MDA5‐positive DM. These autoantibodies may be biomarkers for severe disease and provide insight into disease pathogenesis.

## INTRODUCTION

The novel coronavirus SARS‐CoV‐2 (which causes COVID‐19) has resulted in a pandemic responsible for greater than 175 million infections and 3.7 million deaths worldwide as of June 1, 2021 (1). In many respects, SARS‐CoV‐2 behaves as a typical viral infection, causing symptoms of fever, dyspnea, pharyngitis, and myalgias (2). However, in a subgroup of those infected, SARS‐CoV‐2 leads to a marked inflammatory state with multiorgan failure resulting in high morbidity and mortality.

The SARS‐CoV‐2 viral syndrome has features that overlap with a rare autoimmune syndrome, anti‐melanoma‐differentiation‐associated 5 (MDA5)‐positive dermatomyositis (DM) (3, 4, 5), for example, i) a subgroup of patients with anti‐MDA5‐positive DM present with rapid deterioration of pulmonary function requiring intubation; ii) patients with anti‐MDA5‐positive DM have a unique vasculopathic phenotype, including mucocutaneous ulceration and digital ischemia (6, 7); and iii) patients with SARS‐CoV‐2 and anti‐MDA5‐positive DM with severe disease display similar laboratory and histologic abnormalities, including hyperCKemia, hyperferritinemia, elevated inflammatory cytokine levels, and diffuse alveolar damage (2, 3, 6, 7, 8).

Recently, we reported the presence of anti‐angiotensin converting enzyme‐2 (ACE2) immunoglobulin M (IgM) autoantibodies in patients with SARS‐CoV‐2 with a more severe disease trajectory (8). The autoantibodies induce complement activation on binding to ACE2 and may exert pathogenic effects through binding to endothelial cell ACE2. Given the clinical similarities between SARS‐CoV‐2 and anti‐MDA5‐positive DM, we hypothesized that anti‐MDA5‐positive DM may represent an autoimmune response to a viral infection and suspected that both conditions may be associated with anti‐ACE2 IgM autoantibodies. In the current article, we describe the presence, clinical features, and implications of anti‐ACE2 IgM antibodies in two independent anti‐MDA5‐positive DM cohorts.

## MATERIALS AND METHODS

### Patient cohorts

#### North American DM cohort

Serum samples from 2595 consecutively evaluated patients seen in the Johns Hopkins Myositis Center from 2003 to 2020 were routinely screened by the Euroimmun myositis panel. Of these, 67 were positive for MDA5 antibodies. These were subsequently tested by MDA5 enzyme‐linked immunosorbent assay (ELISA) (MBL); anti‐MDA5 antibodies were confirmed in 52 of 67 serum samples. Only serum samples that were positive by both assays (n = 52) were included in this study. All 52 serum samples were drawn pre‐SARS‐CoV2 pandemic. Fifty of fifty‐two (96%) met probable or definite DM by the 2017 American College of Rheumatology/European League Against Rheumatism (ACR/EULAR) classification criteria (9). The two additional patients had DM characterized by Gottron's sign, interstitial lung disease (ILD), mechanic's hands, and calcinosis. For comparison, a random sampling of 60 additional patients with idiopathic inflammatory myositis (IIM) and Jo‐1 autoantibodies by Euroimmun were studied. Data on demographics and disease characteristics were obtained from the Johns Hopkins Myositis Research Registry, including manual muscle testing, muscle enzymes, pulmonary function testing, chest high‐resolution computed tomography (HRCT) reports, and Cutaneous Dermatomyositis Activity and Severity Index scores (for patients who were lost to follow‐up or died prior to development of the Cutaneous Dermatomyositis Activity and Severity Index, heliotrope sign, Gottron's sign, calcinosis, and cutaneous ulcerations were recorded as discrete variables). Spirometry and lung volume results (forced vital capacity [FVC], total lung capacity, and diffusing capacity of carbon monoxide) are reported as the percentage predicted measured by a test of good or fair quality. Intubation was recorded if attributed to progressive ILD and respiratory failure (eg, elective intubations or intubation for non‐DM causes were excluded). Pneumothorax and pneumomediastinum were considered present only if spontaneous (those post procedure or occurring while patients were on mechanical ventilation were excluded). Rapidly progressive ILD (RP‐ILD) was defined as progressive worsening of dyspnea secondary to ILD requiring hospitalization, supplementary oxygen, or intubation within 3 months of diagnosis of ILD (10). Ulcerations were further divided into ischemic digital ulcers, skin ulcerations, and mucosal or oropharyngeal ulcerations (excluding patients on methotrexate or with suspected herpesvirus). In our center, patients consented under our research registry may choose to provide serum samples at 6‐ to 12‐month intervals for the duration of longitudinal follow‐up. Myositis onset was defined as the first symptom documented in the electronic medical record by the patient, limited to the following: arthralgia, dyspnea, myalgia, weakness, or rash consistent with DM. All patients were called in 2019‐2020 to update their clinical status if they had not been observed longitudinally in clinic. Remission was defined as a patient being off all immunosuppressive and immunomodulatory therapies longer than 1 year while remaining asymptomatic.

#### Japanese DM cohort

Eligible patients with anti‐MDA5 antibodies were selected from the patient database of an autoantibody laboratory at Keio University Hospital from April 2008 to July 2014 and Nippon Medical School Hospital from August 2014 to May 2021. Thirty‐seven patients were positive for anti‐MDA5 antibodies by immunoprecipitation assay followed by confirmatory anti‐MDA5 ELISA. Of these, 32 patients (86%) were included in the present study, in whom the serum samples at diagnosis were available. Twenty‐eight of thirty‐two (87%) were classified as probable or definite DM or amyopathic dermatomyositis (ADM) by the 2017 ACR/EULAR classification criteria. Three additional patients had ADM characterized by typical DM skin rashes, including shawl sign, linear erythema on fingers, and periungual erythema. The remaining patient with rapidly progressive ILD lacked any skeletal muscle or skin involvement. The demographic and clinical data were obtained from the Nippon Medical School Myositis Registry. In addition to the clinical and laboratory parameters used in the Johns Hopkins registry, respiratory status (partial pressure of oxygen [PaO2]/fraction of inspired oxygen [FiO2] ratio, blood oxygen saturation (SpO2)/FiO2 ratio, and alveolar‐arterial oxygen gradient) and serum biomarkers (C‐reactive protein, ferritin, and Krebs von den Lungen‐6) at initial presentation were also recorded.

#### SARS‐CoV‐2 cohort

Hundred and eighteen hospitalized SARS‐CoV‐2 patients were diagnosed using any polymerase chain reaction test with an Emergency Use Authorization from the US Food and Drug Administration. SARS‐CoV‐2 serum samples were from inpatients who had 1) a confirmed diagnosis of SARS‐CoV‐2, 2) survival to death or discharge, and 3) remnant specimens in the Johns Hopkins COVID‐19 Remnant Specimen Biorepository, an opportunity sample that includes 59% of Johns Hopkins Hospital patients with SARS‐CoV‐2 and 66% of patients with lengths of stay of 3 days or more. Serum samples from all patients were assayed; 66 had severe disease (World Health Organization [WHO] ordinal categories 6‐8), and 52 had moderate disease (WHO ordinal categories 3‐5). These studies were approved by the Johns Hopkins Institutional Review Board (IRB) with a waiver of consent because all specimens and clinical data were deidentified by the Core for Clinical Research Data Acquisition of the Johns Hopkins Institute for Clinical and Translational Research. This study was approved by Johns Hopkins (IRB00235356 and IRB00251725) and Nippon Medical School (IRB26‐03‐434) IRBs.

### Autoantibody assays

Anti‐MDA5 autoantibodies were assayed in the North American cohort using Euroimmun line blot myositis panel and MBL ELISA. The Japanese cohort was assayed using MBL ELISA. The Euroimmun and MBL ELISA positive cutoff values were 15 or more and greater than 32, respectively, per manufacturer guidelines. Anti‐MDA5 antibodies were assayed by ELISA (MBL) in the patients with SARS‐CoV‐2. Anti‐ACE2 IgM antibodies were assayed by ELISA as described below and in ref. 8. ELISA plate wells were coated overnight with 50 ng of purified recombinant human ACE2 (Abcam). For each serum sample, two wells were coated with protein (duplicate readout) and an adjacent well was incubated with phosphate‐buffered saline (PBS) to determine background specific to each sample. Wells were washed with PBS plus 0.1% Tween (PBST) and subsequently blocked with 3% milk/PBST. Primary antibody incubations were performed by diluting serum samples 1:200 in 1% milk/PBST overnight at 4°C. Wells were then washed with PBST, followed by incubation with horseradish‐peroxidase‐labeled anti‐human IgM (heavy chain specific; Jackson ImmunoResearch) diluted 1:5000 in 1% milk/PBST (1 hour, room temperature). Color was developed with KPL SureBlue substrate (LGC SeraCare, Milford, MA). Reactions were terminated by adding hydrochloric acid, and absorbances were read at 450 nM. The same anti‐ACE2‐IgM‐positive reference serum sample was included on each plate assayed, and all absorbances were calibrated relative to this reference serum sample. The cutoff for assigning anti‐ACE2 IgM antibody positivity was determined by assaying serum samples from 30 healthy controls. The mean + 3 SDs of these values (0.340 calibrated optical density units) was taken as the cutoff. Immunoglobulin G (IgG) antibodies against ACE2 were assayed and quantitated as previously described (8). Patients positive for anti‐ACE2 IgM were assayed by CoronaChek (Lot# COV20120008; Hangzhou Biotest Biotech Co., Ltd.). Tests were performed per the manufacturer's protocol (8).

### Statistical analysis

Wilcoxon rank sum test and Fisher's exact test were used to compare subgroups and performed using Stata version 14 (StataCorp) and R version 4.1.2 (R Foundation for Statistical Computing).

## RESULTS

### Patient characteristics

Of the 52 North American patients with anti‐MDA5 DM, five (9.6%) were positive for anti‐ACE2 IgM on baseline serum samples. The median time from DM symptom onset to the blood sample assayed for anti‐ACE2 IgM was 1.3 years for the anti‐ACE2‐IgM‐negative group compared with 0.7 years in the anti‐ACE2‐IgM‐positive group (Wilcoxon rank sum test *P* = 0.41). In each case, this was the earliest banked serum sample, obtained at the first patient visit to our clinic. The majority of patients in the North American cohort received corticosteroids prior to serum sample draw for anti‐ACE2 autoantibodies (46 of 52, 88%). Two of the five patients with anti‐ACE2 IgM antibodies had no steroid exposure prior to research blood draw. Compared with the anti‐ACE2‐IgM‐negative group, patients with anti‐ACE2 IgM antibodies were more likely to receive pulse dose methylprednisolone (80% vs 30%, *P* = 0.043) and showed a trend toward higher prevalence of synovitis (100% vs 51%, *P* = 0.059) and worse FVC (median FVC percentage predicted 59% [interquartile range (IQR) 58‐61] vs 78% [IQR 63‐93], *P* = 0.056) (Table 1). When we defined RP‐ILD as progressive worsening of dyspnea secondary to ILD requiring hospitalization, supplementary oxygen, or intubation within 3 months of diagnosis of ILD, three of five patients with anti‐ACE2 IgM (60%) experienced RP‐ILD compared with 9 of 47 (19%) in the anti‐ACE2‐IgM‐negative group (*P* = 0.07).

**TABLE 1:** Characteristics of five North American patients with anti‐MDA5‐positive DM with anti‐ACE2 IgM autoantibodies

	ACE2‐negative (n = 47)	ACE2‐positive (n = 5)	*P*
Patient age at IIM symptom onset, median (IQR)	47.3 (39.7‐54.7)	50.0 (42.3‐51.0)	0.890
Patient age at JH cohort entry, median (IQR)	48.8 (41.1‐55.5)	51.5 (42.6‐53.1)	0.840
Male sex	26%	40%	0.600
Race			0.680
White	49%	40%	
Black	28%	60%	
Asian	6%	0%	
Other	11%	0%	
Declined	2%	0%	
Unknown	4%	0%	
Ethnicity			0.720
Hispanic	2%	0%	
Not Hispanic	85%	100%	
Unknown	13%	0%	
**Duration of follow‐up (years), median (IQR)** *	**3.2 (1.3‐5.7)**	**6.3 (4.8‐9.1)**	**0.045**
Gottron's sign or papules	100%	80%	0.096
Heliotrope sign	72%	100%	0.310
Synovitis on physical examination	51%	100%	0.059
Calcinosis on physical examination	38%	0%	0.150
Proximal CPK (units/L), median (IQR)^a^	49 (38‐102)	84 (58‐87)	0.410
Proximal aldolase (units/L), median (IQR)^a^	7.0 (5.5‐9.1)	8.0 (6.2‐9.4)	0.590
History of malignancy (ever)	11%	20%	0.480
ILD on high‐resolution CT	79%	100%	0.570
Proximal FVC^a^	78 (63‐93)	59 (58‐61)	0.056
Proximal TLC^a^	73 (63‐79)	64 (58‐67)	0.280
Proximal DLCO^a^	68 (48‐84)	59 (51‐83)	0.910
Ulcerations (composite)	66%	80%	1.000
Ischemic digital ulcers on examination or pits	32%	40%	1.000
Mucosal ulcerations (tongue, larynx, vocal cords)^b^	34%	60%	0.340
Cutaneous ulcerations on skin (hands or rest of body)	62%	80%	0.640
Required intubation for rapidly progressive ILD	11%	20%	0.470
Spontaneous pneumothorax or pneumomediastinum	6%	20%	0.340
Proximal muscle weakness	70%	100%	0.310
**Received pulse dose methylprednisolone** *	**30%**	**80%**	**0.043**
Remission	20%	0%	0.570
Death	11%	0%	1.000

*Note*: Race and ethnicity are self‐reported.

Abbreviations: ACE2, angiotensin converting enzyme‐2; CPK, creatine phosphokinase; CT, computed tomography; FVC, forced vital capacity; DLCO, diffusion capacity for carbon monoxide; DM, dermatomyositis; IgM, immunoglobulin M; IIM, idiopathic inflammatory myositis; ILD, interstitial lung disease; IQR, interquartile range; JH, Johns Hopkins; MDA5, melanoma‐differentiation‐associated 5; TLC, total lung capacity.

^a^ Values collected within 1 year of research blood draw for anti‐ACE2 antibodies.

^b^ Not attributable to methotrexate or herpes virus.

* *p* < 0.05.

In the North American cohort, a total of 18 patients received pulse steroids. The indications were as follows: 10 patients received pulse steroids for worsening hypoxemia or progression of ILD, five for worsening cutaneous lesions (anti‐MDA5‐related ulcerations and calcinosis), and three for generalized disease flare, defined as worsening proximal muscle weakness, synovitis, dyspnea, and cutaneous lesions. Of the five patients with anti‐ACE2 IgM autoantibodies, four received pulse steroids. Three of the four patients had an indication of worsening hypoxemia or progression of ILD, whereas the fourth had the indication of severe generalized DM flare.

In the North American cohort, 11 patients (21%) were positive for anti‐ACE2 IgG and three (6%) were positive for both anti‐ACE2 IgM and anti‐ACE2 IgG. Patients with anti‐ACE2 IgG had similar associations to the five patients with anti‐ACE2 IgM. Patients with anti‐ACE2 IgG were more likely to have synovitis (91% vs 46%, *P* = 0.01) and less likely to have calcinosis (9% vs 41%, *P* = 0.07). For the control cohort, we assayed 60 patients with anti‐Jo1‐positive/anti‐MDA5‐negative IIM. Of these, only one patient had borderline/low‐positive anti‐ACE2 IgM antibodies. This patient received pulse dose steroids on disease presentation for a clinical diagnosis of antisynthetase syndrome.

Of the 52 North American patients, 34 had available HRCT images to review with a board‐certified chest radiologist (CTL). Of the five anti‐ACE2‐IgM‐positive patients, two (40%) had a nonfibrotic nonspecific interstitial pneumonia (NSIP) pattern and three (60%) had an organizing pneumonia (OP) pattern. In the 29 patients who were anti‐ACE2‐IgM‐negative with available CT imaging, 3 of 29 (10%) had a nonfibrotic NSIP pattern and 5 of 29 (17%) had OP (*P* = 0.14 and *P* = 0.07, respectively). In the five anti‐ACE2‐IgM‐positive patients in the North American cohort, the average time from diagnosis of ILD (via HRCT) to anti‐ACE2 blood draw was 3 months. All five of these patients (100%) had active ILD on the most proximal HRCT image to blood draw. In contrast, in the anti‐ACE2‐IgM‐negative group, 13 patients had HRCT imaging within 3 months of blood draw and 10 of 13 (77%) had evidence of ILD (*P* = 0.52).

Given the observation that North American anti‐MDA5‐positive and anti‐ACE2‐IgM‐positive patients may have more severe disease and because this cohort is largely outpatient, we considered the possibility that anti‐MDA5‐positive and anti‐ACE2‐IgM‐positive patients experiencing acute decompensation were not captured. We therefore tested a Japanese cohort of 32 patients with anti‐MDA5‐positive DM in which patients and serum samples were captured at disease onset.

In the Japanese cohort, 5 of 32 (15%) patients produced anti‐ACE2 IgM antibodies. Only seven patients (22%) received short courses of corticosteroids prior to the serum sample draw for anti‐ACE2 antibodies. Among the five anti‐ACE2‐IgM‐positive patients, two (40%) had exposure to corticosteroids before the blood sampling. Patients with anti‐ACE2 IgM antibodies had significantly worse oxygenation as defined by a lower PaO2/FiO2 ratio (233 vs 390, Wilcoxon rank sum test *P* = 0.021), a lower SpO2/FiO2 ratio (335 vs 457, *P* = 0.027), and a higher alveolar‐arterial oxygen gradient (91 vs 23 mm Hg, *P* = 0.024) (Table 2). Of the five patients with anti‐ACE2 IgM, three (60%) died within 10 days (IQR 9‐20) of an intensive treatment consisting of pulse dose methylprednisolone followed by high‐dose prednisolone, intravenous cyclophosphamide, tacrolimus, and direct hemoperfusion with polymyxin B‐immobilized fiber (PMX‐DHP)/extracorporeal membranous oxygenation (ECMO), compared with 8 of 27 (29%) in the anti‐ACE2‐IgM‐negative group (*P* = 0.31). Of the anti‐ACE2‐IgM‐positive Japanese patients, the three patients who died had higher anti‐ACE2 IgM antibody levels compared with anti‐ACE2 IgM‐positive survivors (2.33, 1.35, and 1.45 vs 0.81 and 0.72 calibrated optical density units). In contrast to the North American cohort, only 1 of 32 Japanese patients had anti‐ACE2 IgG antibodies, possibly because of a much shorter disease duration in Japanese patients compared with North American patients. This patient was also anti‐ACE2‐IgM‐positive.

**TABLE 2:** Characteristics of five Japanese patients with anti‐MDA5‐positive DM with anti‐ACE2 IgM autoantibodies

	ACE2‐IgM‐negative (n = 27)	ACE2‐IgM‐positive (n = 5)	*P*
Patient age at onset, median (IQR)	52 (44‐64)	59 (52‐66)	0.311
Patient age at first visit, median (IQR)	52 (44‐64)	59 (52‐66)	0.311
Male sex	29%	60%	0.310
Duration of follow‐up (days), median (IQR)	235 (62‐1140)	31 (9‐2050)	0.311
Gottron's sign or papules	88%	80%	0.512
Heliotrope sign	25%	0%	0.560
Synovitis on physical examination	44%	0%	0.130
Calcinosis on physical examination	0%	0%	NA
Proximal CPK,^a^ median (IQR)	109 (76‐260)	152 (114‐663)	0.311
Proximal aldolase,^a^ median (IQR)	8.3 (5.5‐9.6)	10.6 (6.8‐14.6)	0.375
**SpO2/FiO2 ratio, median (IQR)** *	**457 (447‐466)**	**335 (190‐452)**	**0.027**
**PaO2/FiO2 ratio, median (IQR)** *	**390 (320‐426)**	**233 (206‐346)**	**0.021**
**Alveolar‐arterial oxygen gradient, median (IQR)** *	**23.8 (14.2‐41.3)**	**91.0 (33.6‐206)**	**0.024**
CRP (mg/dL), median (IQR)	0.42 (0.06‐1.30)	2.48 (0.54‐3.17)	0.102
Ferritin (ng/mL), median (IQR)	572.2 (244.0‐993.3)	722.3 (572.9‐2630.0)	0.303
KL‐6 (U/mL), median (IQR)	692.0 (466.8‐1173.3)	837.6 (480.0‐1073.0)	0.697
History of malignancy (ever)	7.4%	20%	0.410
ILD on high‐resolution CT	88%	100%	1.000
Proximal FVC,^a^ median (IQR)	80.4 (75.5‐90.3)	71.8 (70.6‐79.9)	0.563
Proximal TLC,^a^ median (IQR)	81.7 (62.8‐100.5)	85.8 (82.2‐89.4)	0.844
Proximal DLCO,^a^ median (IQR)	60.2 (51.1‐85.2)	72.4 (68.4‐76.4)	0.519
Ulcerations (composite)	29%	40%	0.637
Ischemic digital ulcers on examination or pits	0%	0%	NA
Mucosal ulcerations	18%	20%	1.000
Cutaneous ulcerations on skin	11%	20%	0.512
Required intubation for rapidly progressive ILD	7.4%	20%	0.410
Spontaneous pneumothorax or pneumomediastinum	18%	0%	0.564
Proximal muscle weakness on examination	14%	0%	1.000
Received pulse dose methylprednisolone	74%	100%	0.560
Triple immunosuppressive combination regimen, consisting of high‐dose prednisolone, intravenous cyclophosphamide, tacrolimus	66%	80%	1.000
PMX‐DHP/ECMO	14%	60%	0.057
Remission	0%	0%	NA
Death	29%	60%	0.310

Abbreviations: ACE2, angiotensin converting enzyme‐2; CPK, creatine phosphokinase; CRP, C‐reactive protein; CT, computed tomography; DLCO, diffusion capacity for carbon monoxide; DM, dermatomyositis; ECMO, extracorporeal membrane oxygenation; FiO2, fraction of inspired oxygen; FVC, forced vital capacity; IgM, immunoglobulin M; ILD, interstitial lung disease; IQR, interquartile range; KL‐6, Krebs von den Lungen 6; MDA5, melanoma‐differentiation‐associated 5; NA, not applicable; PaO2, partial pressure of oxygen; PMX‐DHP, polymyxin B immobilized fiber column direct hemoperfusion; SpO2, oxygen saturation; TLC, total lung capacity.

^a^ Values closest to the time of blood draw for anti‐ACE2 antibodies.

* *p* < 0.05.

All 10 anti‐ACE2‐IgM‐positive patients (both in North America and Japan) were assayed by CoronaChek. All 10 serum samples were negative for both IgM and IgG SARS‐CoV‐2 antibodies. Additional data on line blot results for both cohorts can be found in Supplementary Tables 1 and 2.

### Serial anti‐ACE2 antibody titers

For all anti‐MDA5‐positive serum samples, the initial screen for anti‐ACE2 IgM and IgG antibodies was done on the first available serum sample. The availability of banked serial samples for 27 of 52 North American patients and 20 of 32 Japanese patients enabled us to test samples over time. In both cohorts, no patient became anti‐ACE2‐IgM‐positive if they were initially negative for these antibodies. Furthermore, in three anti‐ACE2‐IgM‐positive patients with banked serial samples (taken 1, 3, and 11 months after the first sample), anti‐ACE2 IgM antibodies were absent in the second sample, whereas anti‐MDA5 antibody titers remained robust. Two of these three patients also had anti‐ACE2 IgG present on the first bleed and subsequently became anti‐ACE2‐IgG‐negative.

### MDA5 autoantibodies in SARS‐CoV‐2 patients

Given the similarities between anti‐MDA5‐positive DM and SARS‐CoV‐2, we also tested patients with SARS‐CoV‐2 for autoantibodies to anti‐MDA5. We tested 118 patients with SARS‐CoV‐2 with a spectrum of clinical severity by ELISA for MDA5 antibodies; none were positive. Notably, this includes 18 patients with SARS‐CoV‐2 in whom anti‐ACE2 IgM autoantibodies were detected.

## DISCUSSION

We describe the presence of anti‐ACE2 IgM antibodies in two separate anti‐MDA5‐positive DM cohorts: approximately 10% (n = 5 of 52) in a largely outpatient North American cohort and 15% (n = 5 of 32) in a Japanese cohort enriched for new‐onset or severe disease. Although the number of patients positive for anti‐ACE2 IgM is small, the available data suggest their presence may be a marker of disease severity. In the initial exploration of our North American population, we observed that all five anti‐ACE2‐IgM‐positive patients experienced proximal muscle weakness and ILD. Furthermore, patients with anti‐ACE2 IgM experienced more inflammatory arthritis, RP‐ILD, and worse FVC, all of which approached statistical significance (*P* ~ 0.05) despite the small number of patients. This observation, in the context of anti‐ACE2 IgM associating with severe disease in patients with SARS‐CoV‐2 (8), prompted us to investigate a Japanese anti‐MDA5‐positive DM cohort in which data were available from disease onset. Strikingly, all five patients with anti‐ACE2 IgM in the Japanese cohort required intensive immunosuppressive regimens and suffered significantly worse oxygenation, ultimately leading to death in three of five (60%) patients.

Although we report a 10% prevalence of anti‐ACE2 IgM in the North American cohort, the true prevalence is unknown given the observations that i) anti‐ACE2 IgM disappears over time, ii) the time between DM symptom onset and first research blood draw in our cohort was 1.3 years in the anti‐ACE2‐IgM‐negative group, and iii) the effect of immunosuppression on anti‐ACE2 IgM titers is unknown. Additional studies performed on anti‐MDA5‐positive DM cohorts with short intervals between symptom onset and blood draw (such as the Japanese cohort) are warranted. These will likely provide important insights into understanding the extent to which anti‐ACE2 IgM is present.

The presence of anti‐ACE2 IgM and IgG raises the question of whether these antibodies are markers of tissue damage, are reflective of generalized immune activation, or are actually involved in disease initiation and/or propagation. Excluding anti‐Ro52, only 2 of 10 anti‐ACE2‐IgM‐positive patients produced additional autoantibodies on line blot, suggesting that a state of generalized immune activation responsible for anti‐ACE2 IgM may be less likely (Supplementary Tables 1 and 2). Casciola‐Rosen et al (8) recently reported 18 of 66 (27%) patients with severe SARS‐CoV‐2 produced anti‐ACE2 IgM and demonstrated that these IgM antibodies activate the complement pathway. Because ACE2 is expressed at the surface of endothelial cells, such binding might induce endothelial damage (11, 12).

In our previous work (8), we demonstrated that patients with other infectious and rheumatic diseases did not produce anti‐ACE2 IgM. In the present study, among our comparator anti‐Jo1‐positive IIM cohort, only 1 of 60 patients had low‐positive anti‐ACE2 IgM antibodies. Similar to the five North American anti‐MDA5‐positive and anti‐ACE2‐IgM‐positive patients, this single anti‐Jo1‐positive patient had ILD and inflammatory arthritis. Noting that anti‐ACE2 IgM antibodies appear to decrease over time, further studies of larger cohorts with serum samples drawn proximal to rheumatic disease symptom onset are warranted.

The presence of anti‐ACE2 IgM in a subgroup of patients with both anti‐MDA5‐positive DM and SARS‐CoV‐2 may provide insight into the etiology of anti‐MDA5‐positive DM. Both have subgroups that behave similarly: initial viral symptoms that deteriorate into respiratory failure with marked inflammation and superimposed features of autoimmunity, including vasculitis and vasculopathy. It is conceivable that anti‐MDA5‐positive DM may represent an autoimmune response to a viral infection. In this regard, it is intriguing that the antigenic target of anti‐MDA5‐positive DM (MDA5) is a pattern recognition receptor that detects RNA viruses and initiates the interferon response (13). Furthermore, SARS‐CoV‐2 has been demonstrated to elicit interferon production via MDA5‐mediated sensing (14). Interestingly, in the North American cohort, DM symptom onset in 9 of 11 patients with anti‐ACE2 antibodies was in September to February. A similar seasonal association has recently been published in a Japanese anti‐MDA5‐positive DM population (15). This is noteworthy because during this season, several human RNA viruses circulate, including influenza, respiratory syncytial virus, rhinoviruses, and coronaviruses. One coronavirus in particular, HCoV‐NL63, also binds to the ACE2 receptor and is frequent in the human population (16). Further studies quantifying immune responses against the virome in patients with anti‐MDA5‐positive DM are warranted to address the hypothesis that RNA viruses initiate autoimmunity in this disease spectrum.

Given the phenotypic similarities between SARS‐CoV‐2 and anti‐MDA5‐positive DM, we and others have investigated the presence of anti‐MDA5 IgG antibodies in patients with SARS‐CoV‐2 (17). Liu et al (17) reported an anti‐MDA‐5 autoantibody prevalence of 132 of 274 (48%) in patients with SARS‐CoV‐2 using a cutoff of 5 units/ml (MBL ELISA). Using the same assay with the manufacturer recommended cutoff of greater than 32 to define anti‐MDA5 positivity, we did not detect anti‐MDA5 autoantibodies in any patients with SARS‐CoV‐2 patients. These differences in results are likely explained by the cutoffs chosen to define positivity. Anti‐MDA5 antibody positivity was not a feature of patients infected with SARS‐CoV‐2 in our North American cohort.

We have described for the first time the prevalence and clinical phenotype of anti‐ACE2 IgM autoantibodies in anti‐MDA5‐positive DM. This presence of this autoantibody may be a biomarker for severe disease and provide insight into disease pathogenesis.

## AUTHOR CONTRIBUTIONS

All authors were involved in drafting the article or revising it critically for important intellectual content, and all authors approved the final version to be published. Dr. Mecoli had full access to all of the data in the study and takes full responsibility for the integrity of the data and accuracy of the data analysis.

### Study conception and design

Mecoli, Rosen, Christopher‐Stine, Casciola‐Rosen.

### Acquisition of data

Mecoli, Yoshida, Paik, Lin, Danoff, Hanaoka, Rosen, Christopher‐Stine, Kuwana, Casciola‐Rosen.

### Analysis and interpretation of data

Mecoli, Yoshida, Paik, Lin, Danoff, Hanaoka, Rosen, Christopher‐Stine, Kuwana, Casciola‐Rosen.

## Supporting information


**Disclosureform:** 
